# Crop Domestication Alters Floral Reward Chemistry With Potential Consequences for Pollinator Health

**DOI:** 10.3389/fpls.2018.01357

**Published:** 2018-09-26

**Authors:** Paul A. Egan, Lynn S. Adler, Rebecca E. Irwin, Iain W. Farrell, Evan C. Palmer-Young, Philip C. Stevenson

**Affiliations:** ^1^Department of Plant Protection Biology, Swedish University of Agricultural Sciences, Alnarp, Sweden; ^2^Department of Biology, University of Massachusetts Amherst, Amherst, MA, United States; ^3^Department of Applied Ecology, North Carolina State University, Raleigh, NC, United States; ^4^Royal Botanic Gardens, Kew, Richmond, United Kingdom; ^5^Natural Resources Institute, University of Greenwich, London, United Kingdom

**Keywords:** domestication, floral rewards, *Vaccinium*, crop evolution, pollinator-pathogen interactions, *Bombus impatiens*, pollinator health, phytochemicals

## Abstract

Crop domestication can lead to weakened expression of plant defences, with repercussions for herbivore and pathogen susceptibility. However, little is known about how domestication alters traits that mediate other important ecological interactions in crops, such as pollination. Secondary metabolites, which underpin many defence responses in plants, also occur widely in nectar and pollen and influence plant-pollinator interactions. Thus, domestication may also affect secondary compounds in floral rewards, with potential consequences for pollinators. To test this hypothesis, we chemically analysed nectar and pollen from wild and cultivated plants of highbush blueberry (*Vaccinium corymbosum* L.), before conducting an artificial diet bioassay to examine pollinator-pathogen interactions. Our results indicated that domestication has significantly altered the chemical composition of *V. corymbosum* nectar and pollen, and reduced pollen chemical diversity in cultivated plants. Of 20 plant metabolites identified in floral rewards, 13 differed significantly between wild and cultivated plants, with a majority showing positive associations with wild compared to cultivated plants. These included the amino acid phenylalanine (4.5 times higher in wild nectar, 11 times higher in wild pollen), a known bee phagostimulant and essential nutrient; and the antimicrobial caffeic acid ester 4-*O*-caffeoylshikimic acid (two times higher in wild nectar). We assessed the possible biological relevance of variation in caffeic acid esters in bioassays, using the commercially available 3-*O*-caffeoylquinic acid. This compound reduced *Bombus impatiens* infection by a prominent gut pathogen (*Crithidia*) at concentrations that occurred in wild but not cultivated plants, suggesting that domestication may influence floral traits with consequences for bee health. Appreciable levels of genetic variation and heritability were found for most floral reward chemical traits, indicating good potential for selective breeding. Our study provides the first assessment of plant domestication effects on floral reward chemistry and its potential repercussions for pollinator health. Given the central importance of pollinators for agriculture, we discuss the need to extend such investigations to pollinator-dependent crops more generally and elaborate on future research directions to ascertain wider trends, consequences for pollinators, mechanisms, and breeding solutions.

## Introduction

Humans have domesticated and selectively bred crops for millennia (Smith, 2001). While domestication has delivered many agronomic benefits, including enhanced yield and other desirable traits (Evans, 1996; Bai and Lindhout, 2007; Dempewolf et al., 2017), negative or unintended consequences of this process have included reduced genetic diversity (Van de Wouw et al., 2010; Meyer et al., 2012), and the impaired function or disappearance of potentially useful traits (Rosenthal and Dirzo, 1997; Li et al., 2017) such as physical or chemical defences against herbivory and disease (Wink, 1988; Jones, 1998; Gols et al., 2008; Sujana et al., 2012). Plant defence traits can impose considerable resource allocation costs, and can thereby trade-off with fitness or yield (Kempel et al., 2011). Hence, where selection is consistently imposed over many generations to maximise crop yield, agronomic gains may accrue at the risk of compromising other traits from which resources are diverted (Rosenthal and Dirzo, 1997; Brown and Rant, 2013). It is only relatively recently, however, that the extent of domestication impacts on crop ecological function has come to be fully appreciated (Meyer et al., 2012; Chen et al., 2015; Whitehead et al., 2017). This topic has garnered increased attention due to growing interest in the agronomic potential of traits traditionally neglected in crops, such as herbivore-induced plant volatiles (HIPVs) and extrafloral nectaries involved in the attraction of pest natural enemies (Rasmann et al., 2005; Rodriguez-Saona et al., 2009; Chen et al., 2015; Heil, 2015; Li et al., 2017).

In contrast to impacts on crop interactions with herbivores and pathogens, very little is known about the effect of domestication on interactions with pollinators and the traits which mediate these interactions. What little is known on this topic to date has mostly come from the study of ornamental plants, where widespread loss of floral scent is a reported consequence of breeding (Vainstein et al., 2001; Pichersky and Dudareva, 2007). This lack of information on domestication effects on pollinator-relevant traits, including floral reward chemistry, is surprising, given the importance of insect pollination for agriculture. About 75% of the world’s staple food crops rely to some extent on the ecosystem service of pollination (Klein et al., 2007), which contributes an estimated $351 billion (USD) annually to global food production (Lautenbach et al., 2012). Pollinators also have the potential to contribute to human nutritional health and need for micronutrients (Chaplin-Kramer et al., 2014; Ellis et al., 2015), and improve global food security (Bailes et al., 2015).

Although historically studied separately, plant-herbivore and plant-pollinator interactions are interconnected (Strauss and Armbruster, 1997; Adler et al., 2001; Irwin et al., 2003; Strauss and Irwin, 2004; Kessler and Halitschke, 2009; Lucas-Barbosa, 2016; Muola et al., 2017; Nepi et al., 2018). For example, a broad range of defence-related secondary metabolites (including alkaloids, phenolics, and cyanogenic glycosides) are expressed in floral rewards, including nectar and pollen (Baker, 1977; Rhoades and Bergdahl, 1981; Adler, 2000; Adler and Irwin, 2005; Heil, 2011; Arnold et al., 2014; Irwin et al., 2014; Stevenson et al., 2017). These compounds range widely in concentration – from trace amounts, to concentrations similar to other plant tissues (Stevenson et al., 2017, and references therein) – and in ecological function. The ecological effects of secondary compounds in floral rewards include warding off nectar robbers and microbial growth (Irwin et al., 2004; González-Teuber and Heil, 2009; Barlow et al., 2017), enhancing pollinator memory (Wright et al., 2013), and reducing pollinator infection by pathogens and parasites (Baracchi et al., 2015; Richardson et al., 2015; Erler and Moritz, 2016; Palmer-Young et al., 2016). Evidence now suggests that secondary metabolites occur ubiquitously in floral rewards, and commonly show phenotypic integration across herbivore- and pollinator-consumed materials in wild and cultivated plants (Adler et al., 2006, 2012; Kessler and Halitschke, 2009; Manson et al., 2012; Cook et al., 2013; Richardson et al., 2016; Palmer-Young et al., in press). It is therefore of great interest to assess whether domestication effects on plant defence are also mirrored for floral reward chemistry, with potential ramifications for ecological function.

We used blueberry as a crop system to examine the effect of domestication on floral reward chemistry and its potential consequence for pollinators. We included in this study wild and cultivated plants of *Vaccinium corymbosum* L. (highbush blueberry; Ericaceae), including cultivated hybrid and introgressed crosses with *V. angustifolium* Ait. (lowbush blueberry). The relatively recent and well-documented history of domestication of *Vaccinium* species (including cranberry, lingonberry, and bilberry) has rendered the genus a useful model for the study of domestication effects (Rodriguez-Saona et al., 2011; Song and Hancock, 2011; Rivera et al., 2015, 2016; Hernandez-Cumplido et al., 2018). The history of domestication of highbush and lowbush blueberry dates back to 1908 and 1909, respectively, when the first wild plants were collected from Greenfield, New Hampshire, United States, for cultivation and selective breeding (Gough, 1993). This initial highbush collection, together with two additional wild selections from New Jersey (Ehlenfeldt, 2009), formed some of the first early cultivars of *V. corymbosum* (‘Brooks,’ ‘Sooy,’ ‘Rubel’). Together with their crosses (‘Katherine,’ ‘Pioneer’), these first selections continue to make the largest genetic contributions to modern highbush cultivars (Ehlenfeldt, 1994; Lobos and Hancock, 2015), which form the basis of the global blueberry industry.

We examined nectar and pollen chemistry in eight cultivars or interspecific crosses of *V. corymbosum*, and as a basis for comparison, sampled from three wild populations within ca. 40 km of where the original founding cultivar ‘Brooks’ was collected. To examine potential domestication effects on pollinator health via changes in the pathogens of pollinators, we conducted an artificial diet bioassay with the common eastern bumble bee, *Bombus impatiens* Cresson. Bees were infected with the pathogen *Crithidia*, before manipulating dietary levels of a caffeic acid ester at concentrations representative of wild and cultivated plant nectar to compare their effect on infection. C*rithidia* species are Trypanosomatid gut pathogens that infect *Bombus* species via faecal-oral contamination (Durrer and Schmid-Hempel, 1994). Infection by *Crithidia* can reduce learning (Gegear et al., 2005, 2006), colony growth rates (Shykoff and Schmid-Hempel, 1991) and queen founding success (Brown et al., 2003), and is associated with reduced reproduction in wild bumble bee colonies (Goulson et al., 2018). In Massachusetts, United States *Crithidia* can infect up to 80% of *Bombus* (Gillespie, 2010).

Specifically, we tested the following hypotheses: (1) Domestication has altered chemical composition and reduced chemical diversity of nectar and pollen; (2) Nectar and pollen chemical traits show genetic variation and heritability, but cultivars are phenotypically less variable than wild populations, consistent with their clonal propagation; (3) Nectar and pollen chemical concentration and composition are genetically correlated, which could constrain targeted breeding for floral reward chemistry; and (4) Altered expression of nectar antimicrobial compounds, such as caffeic acid esters, can influence pollinator-pathogen interactions.

## Materials and Methods

### Plant Material and Field Sampling

Eight cultivars were selected for inclusion in this study, based on suitable replication across sampled farms: ‘Patriot’ (P), ‘Reka’ (R), ‘Liberty’ (L), ‘Bonus’ (B), ‘Friendship’ (F), ‘Northland’ (N), ‘BlueCrop’ (BC), and ‘Spartan’ (S). The genetic origin of the majority of these cultivars is pure *V. corymbosum* (cultivars B, L, N, R, and S), whereas cultivars BC, F, P are interspecific hybrids with minor parentage from *V. angustifolium* (**Supplementary Table 1**). A range of ploidy levels are known to exist within the genus *Vaccinium*. However, commercially grown highbush cultivars, including those with minor percentages of *V. angustifolium*, are reported to be tetraploids (Rowland and Hammerschlag, 2005). The cultivars included in this study, and domesticated highbush plants generally, are typically propagated by vegetative means (softwood or hardwood cuttings) (Gough, 1993), meaning that all sampled plant individuals of the same cultivar were genetic clones.

Nectar and pollen from wild and cultivated highbush blueberry were sampled in western Massachusetts, United States, in May 2014. Wild samples were collected from three sites at least 5 km apart between May 15 to 26 [Harvard Forest (HF): 42°32′6″N, 72°11′19″W, Harvard Pond (HP): 42°29′54″N, 72°12′47″W, and Quabbin Reservoir (Q): 42°23′32″N, 72°24′11″W]. We sampled cultivars at several farms between May 19 and 28 from Cold Spring Orchard (42°15′6″N, 72°21′38″W; cultivars P, R, L, B, F, and N), Kenburn Orchard (42°36′43″N, 72°39′18″W; cultivar P), Nourse Farms (42°25′49″N, 72°35′18″W; cultivars BC and S) and Sobieski’s River Valley Farm (42°27′12″N, 72°35′43″W; cultivars BC and S). To ensure no large confounding effect of sampling across multiple farms, cultivar-by-farm interactions were tested but were not significant (data not shown) for nectar and pollen chemical composition and diversity, as quantified below. For analyses other than nectar and pollen chemical composition and diversity, we included only cultivars which had been sampled from a single farm (Cold Spring Orchard).

Ten wild plants were sampled per site, and 5 cultivated plants per cultivar per farm. To prevent pollinator access to nectar, flowers were bagged with mesh usually one day before collection for all plants except at Sobieski’s farm, where we obtained sufficient nectar without bagging; sometimes bags were left on plants for several days if weather was not conducive to collection (e.g., rain). Where a mixture of bagged and unbagged samples were collected for a cultivar (i.e., BC and S), we tested to affirm that bagging did not hold a significant effect on compound concentrations (data not shown). Nectar was typically collected from flowers using 2 μl glass microcapillary tubes inserted into the flower. Nectar was pooled across flowers within plants with a target of 20 μl per plant (range: 6.76–32.16 μl), and added to 80 μl ethanol in a 1.5 ml microcentrifuge tube. Anthers were collected using forceps, and thus our samples included the pollen sac and a small amount of filament in addition to granular pollen. We collected at least 5 mg per plant by pooling across flowers within plants. Nectar and pollen samples were placed on ice in a cooler in the field, frozen at -20°C, and then freeze-dried prior to analysis.

### Chemical Analysis

Freeze-dried nectar was redissolved in 50 μL MeOH. For extraction of pollen, dried samples were sonicated for 10 min in 1 ml MeOH, and incubated at room temperature for 24 h. Samples were then centrifuged at 13,000 rpm for 5 min, and supernatant transferred to glass vials for analysis. Sample analyses were performed by liquid chromatography electrospray ionisation mass spectroscopy (LC-ESIMS) and UV spectroscopy using a Micromass ZQ LC-MS (Waters, Elstree, Herts, United Kingdom). Aliquots of nectar were injected directly onto a Phenomenex (Macclesfield, Cheshire, United Kingdom) Luna C18(2) columns (150 × 3.0 mm i.d., 5 um particle size) and compounds eluted using MeOH (A), H_2_O (B) and formic Acid (C) with A = 0%, B = 90% at T = 0 min; A = 90%, B = 0% at T = 20 min and held for 10 min with C at 10% throughout the analyses. Column temperature was 30°C with flow rate = 0.5 ml min^-1^. High resolution MS spectra were used to provide additional data for compound identification and were recorded for a subset of samples using a Thermo LTQ-Orbitrap XL mass spectrometer (Waltham, MA, United States) with compound separation on an Accela LC system using similar elution parameters as described above. Except for identification of caffeoylquininc and caffeoylshikimic acids (see below), compounds were identified by comparison with mass spectra available via the NIST spectral database version 2.0 and with authentic standards available at Royal Botanic Gardens, Kew. Compounds were quantified using calibration curves with authentic standards based on UV absorbance or compound ionisation in the mass spectrometer. Where these were not available, compound quantities were determined using standard curves calculated from the UV absorbance of compounds with the same chromphore.

These methods also permitted the detection of two amino acids, phenylalanine and tryptophan. For convenience, we used the term ‘total amino acids’ to refer to the two amino acids only detected and quantified. Compound concentrations are for nectar expressed in μmol L^-1^ original volume, and for pollen in μmol kg^-1^ dry mass. We refer to 3-, 4-, and 5-*O*-caffeoylquinic acids (CQAs) collectively as chlorogenic acids, and specifically to 3-CQA as chlorogenic acid.

Our LC-MS analysis identified CQAs as principal secondary metabolites in nectar based on their mass spectral properties, which were similar to those reported previously for these compounds (Schütz et al., 2004). This identification was supported by comparison with authentic standards from our own in-house (Jodrell Laboratory) collection, and their accurate mass pseudomolecular ion of *m/z* 355.1038 which gave a protonated molecular formula [M+H]^+^ of C_16_H_19_O_9_. CQAs are esters of caffeic acid with a hydroxylated cyclohexanoic acid, quinic acid (Schütz et al., 2004). Our analysis also identified an additional caffeic acid ester in nectar of *V. corymbosum* which had a similar UV spectrum to the CQAs (indicative of a caffeoyl moiety). However, this ester recorded a pseudomolecular ion of *m/z* 337.0926 (calcd. 337.0918), corresponding to a protonated molecular formula [M+H]^+^ of C_16_H_17_O_8_, suggesting a dehydro derivative of CQA. Further comparison of the (-) MS2 data showed major fragment ions at *m/z* 291 and 179, indicative of caffeoylshikimic acids as reported in *Phoenix dactylifera* (Habib et al., 2014). Subsequent co-elution with an authentic laboratory standard and (-) MS2 showing similar fragments at 179, 135, 161, and 291 allowed assignment of the compound to 4-*O*-caffeoylshikimic acid (4-CSA).

### Bumblebee Infection Assay

As per our chemical analysis, 4-CSA largely differentiated wild and cultivated plants of *V. corymbosum* (see section “Results”), and thus warranted further biological evaluation. We tested chlorogenic acid (3-CQA) as a proxy for this compound, since no commercial source of 4-CSA was available, and different caffeic acid esters typically show equivalent biological activities (Guzman, 2014), likely owing to their common *ortho*-dihydroxy substitution (Stevenson et al., 1993). For example, in bioassays against various species of bacteria and yeast, 3-CQA, 5-CQA, and other caffeic acid esters frequently possessed identical minimum inhibitory concentration (MIC) values (Zhu et al., 2004; Xia et al., 2011; Guzman, 2014).

Bioassays were conducted with workers of *Bombus impatiens* parasitized with *Crithidia*. To test how 3-CQA affected *Crithidia* infection, we experimentally infected *Bombus impatiens* workers before feeding them 30% sucrose solution without or with 3-CQA (C_16_H_18_O_9_; molecular weight 354.31 g/mol; CAS number 327-97-9; Sigma-Aldrich, St. Louis, MO, United States) at two concentrations, low (2.8 μM [1 ppm] 3-CQA dissolved in 30% sucrose solution), and high (56.5 μM [20 ppm] dissolved in 30% sucrose solution). These represented low and high concentrations of 4-CSA found naturally in cultivated (4.44 μM ± 7.52 SD) and wild (22.03 μM ± 27.46 SD) plants, respectively (see section “Results”).

Thirty worker bees were tested per treatment (*n* = 90 bees total), evenly spread across three source colonies (Biobest Canada, Leamington, Canada; 10 bees per source colony per treatment). Bees were removed from their source colonies, randomly assigned to treatments, starved for 4–6 h, and then experimentally infected with *Crithidia* on the same day. *Crithidia* were isolated from wild *B. impatiens* collected in Massachusetts, United States (GPS coordinates: 42°24′25″N, 72°31′46″W), and maintained in *B. impatiens* colonies in the laboratory. *Crithidia* inoculum was prepared according to a standard protocol (Richardson et al., 2015). In brief, on the day of experimental inoculation, 10 workers were sacrificed from the infected colonies and their intestinal tracts ground individually in 300 μl of dH_2_0. After vortexing the samples, we allowed them to settle at room temperature for 4 h. We then placed 10 μl of the clear solution on a haemocytometer and examined it for *Crithidia*. For samples that contained *Crithidia*, we transferred 50–150 μl of solution to a clean container and diluted to make an approx. 30% sucrose solution with 6000 *Crithidia* cells × 10 μl^-1^. Each bee was provisioned with 10 μl of inoculum containing 6000 *Crithidia* cells, which is within the range of concentrations of the pathogen that bees encounter (e.g., Schmid-Hempel and Schmid-Hempel, 1993; Otterstatter and Thomson, 2006). Immediately following inoculation, bees were housed individually in plastic containers. They were given one lump of honey bee collected pollen (Koppert Biological Systems, Howell, MI, United States) mixed with sugar water, and new sugar solution *ad libitum* of the appropriate treatment. Pollen and nectar were replaced daily for 7 days.

After 7 days, we sacrificed individuals and prepared intestinal tracts as when making *Crithidia* inoculum. We placed 10 μl of intestinal solution on a haemocytometer and counted the number of *Crithidia* cells in five subfields of the grid at 10x magnification, totalling 0.02 μl, with a dissecting microscope. We summed these counts and calculated the number of *Crithidia* cells per mL for each bee. We also removed the right forewing of each bee, mounted it on a microscope slide, and measured the radial cell length as an estimate of bee size (Harder, 1982).

### Statistical Analyses

#### Chemical Composition

Comparisons of chemical composition between wild and cultivated plant nectar and pollen were made on both an absolute and proportional basis, which describe two different aspects of composition. Hence, the quantitative concentration of compounds was used directly in the absolute composition analysis, whereas the relative concentration of compounds (their percent contribution on a molar basis relative to other compounds in the sample) was used in proportional composition analysis. For this, a proportional dataset was generated in which compound concentrations within a sample row were standardised by dividing each by the row total. Absolute composition was examined by plotting principle components analysis (PCA) ordinations using the ‘rda’ function in the R package ‘vegan’ (Oksanen et al., 2017). PCA provided good representation of the multivariate composition of nectar and pollen, in which the cumulative proportion of variance explained by the first two components was 0.84 and 0.65, respectively. For proportional analyses, unconstrained NMDS (Non-metric multidimensional scaling) ordinations were fitted and plotted based on a Bray-Curtis distance metric in ‘vegan.’

As a complement to ordinations, the hypothesis that wild and cultivated plants differed in nectar and pollen chemical composition was explicitly tested using permutational multivariate ANOVAs (function ‘adonis’ in vegan). For this, distance matrices were first generated for absolute composition based on Euclidean distance (to better preserve quantitative relationships), and for proportional composition using Bray–Curtis distance. Similar to univariate ANOVA, this technique relies on the assumption of multivariate homogeneity between groups (i.e., between wild and cultivated plants). According to homogeneity of multivariate dispersions tests (vegan function ‘betadisper’), this assumption was met for all variables except nectar proportional composition. As an alternative to permutational multivariate ANOVA, we therefore opted to compare nectar proportional composition based on axis scores extracted from NMDS ordination. NMDS axis 1 and 2 scores provided a reduced dimensionality representation of proportional composition, and were each fitted as a univariate response in generalised linear mixed models (GLMMs) using the R package ‘nlme’ (Pinheiro et al., 2017). The GLMMs were fitted with an explanatory variable of ‘origin’ (wild or cultivated), and a nested random effect of group ID (cultivar or population name). A variance structure was added to both models via the ‘weights’ function of nlme, to control for unequal variances between wild and cultivated plants. Models were otherwise validated by examining standardised residuals for normal distribution.

‘Indicator compound analysis’ (or multilevel pattern analysis) was implemented using R package ‘indicspecies’ (Cáceres and Legendre, 2009) to further probe any potential differences in chemical composition between wild and cultivated plants. This analysis used a permutational approach to test for compound association (or differences in abundance) between different groups (De Cáceres, 2013), in our case wild and cultivated plants. Compound association values (i.e., point-biserial correlation coefficients) and their corresponding *p*-values (testing the null hypothesis that the correlation is zero) were generated using the ‘multipatt’ function, based on 999 permutations, and use of a corrected correlation coefficient (specified as ‘r.g’ in multipatt). This correlation coefficient is similar to Pearson correlation, and is likewise bounded between zero and one.

#### Chemical Diversity

Chemical diversity was assessed both in terms of compound richness (the total number of compounds per sample), and through calculation of a chemical diversity index (H′_C_) per sample, following previous authors (Epps et al., 2007; Meier and Bowman, 2008). Calculation of this index used the same equation as for Shannon–Wiener diversity (computed using the vegan ‘diversity’ function). Hence, H′_C_ took into account information on compound concentration as well as compound richness. We used linear mixed models (LMMs) to examine potential differences in H′_C_ between wild and cultivated plants for nectar and pollen. LMMs were fitted using the nlme package, specifying H′_C_ as a response, ‘origin’ (cultivated or wild) as a fixed effect, and group ID (cultivar or population name) as a nested random effect. We used ANOVAs to test for significant differences in H′_C_ between cultivars, as well as between wild populations. All models were validated by checking residuals for equal variance and normal distribution.

#### Genetic Variation, Correlation and Heritability

We examined genetic variation, genetic correlation, and heritability (as described below) for the six cultivars sampled at Cold Spring Orchard for six traits summarising nectar and pollen chemistry: chemical diversity (H′_C_), chemical composition (i.e., NMDS axis 1 scores), total amino acids, total phenolic acids, total flavonols, and total flavan-3-ols. The latter were only included for pollen analyses, as these compounds were not identified in nectar. Chemical composition was quantified via NMDS, as described above. Since our estimates of genetic variation (*V_G_*) and covariance (*Cov_G_*) were based on the use of clones, which therefore included both additive and other genetic effects, these are considered total genetic estimates (de Araújo and Coulman, 2004). We hence report estimates of total genetic variation, total genetic correlation, and broad-sense (or clonal) heritability (H^2^) – the genetic contribution to cultivar phenotypic variance. High values of H^2^ hence indicate that a focal trait is largely under genetic control, whereas low values mean that most phenotypic variance is due to environmental factors.

Genetic variation and heritability were analysed by fitting variance component (or random effects) models using the R packages ‘rptR’ (Stoffel et al., 2017), ‘lme4,’ and its extension ‘lmerTest’ (Kuznetsova et al., 2017). Each trait was fitted as a univariate response, and ‘cultivar’ as a random effect. We used the ‘rand’ function in lmerTest to assess the magnitude and significance of genetic variation, based on the chi-squared (*χ*^2^) statistic, and its corresponding *p*-value from a likelihood ratio test. From the same model, the ‘rpt’ function in rptR was used to provide estimates of H^2^ and its standard error. A bootstrapping procedure (*n* = 1000 iterations) was implemented within the function to test significance for H^2^.

Genetic correlations between traits were analysed using Bayesian multivariate mixed models, implemented in the package ‘MCMCglmm’ (Hadfield, 2010). For within-material correlations (e.g., between total amino acids and total flavonols), two models were fitted; one which included all nectar traits as a multivariate response, and a second including all pollen traits. For between-material correlations (e.g., between total amino acids across nectar and pollen), a combined model was fitted which included all nectar and pollen traits as a multivariate response. Use of multivariate models in this sense allowed genetic variance (*VG*) and covariance (*Cov_G_*) components to be estimated for and between traits, respectively. Based on these components, genetic correlation (rG) was calculated for a given set of traits, A and B, as: rG = *Cov_G_*(A,B)/sqrt[*V_G_*(A), *V_G_*(B)]. Following Houslay and Wilson (2017), we reported the posterior mean of rG estimates, and assessed the statistical support for rG through plotting 95% credible intervals for the posterior distribution. When this interval did not include zero, we considered genetic correlations to be statistically supported. All traits were fitted as a Gaussian response, aside from nectar total amino acids and total phenolic acids, for which an ‘exponential’ family was used. For each model, we employed uninformative parameter-expanded priors for the random effects, and allowed for different trait means, unstructured (co)variances, and trait-specific intercepts. The within-material and between-material models were run for 160,000/1,500,000 iterations, respectively, discarding the first 10,000 iterations as burn-in, and sampling every 75 iterations thereafter. Convergence was assessed by means of the Gelman-Rubin criterion, in which four chains were run to check that they converged to the same posterior distribution. As additional diagnostics, we examined trace and autocorrelation plots for consistent variation and non-autocorrelation between iterations, respectively, and checked that posterior estimates were robust to different starting priors.

As an alternative to direct comparisons of genetic variation in traits between wild and cultivated plants (which was not possible since we have no basis to partition genetic variation from phenotypic variation in wild plants), we compared the phenotypic variability of nectar and pollen composition. Phenotypic variability in chemical composition was measured as the Euclidean distance (or dispersion) of plant individuals from their respective cultivar/population multivariate centroid, following the similar use of this approach in community ecology (Anderson et al., 2006). Hence, to examine whether phenotypic variability differed between wild and cultivated plants, we extracted individual plant distances to their respective group centroids for both nectar and pollen (using the ‘betadisper’ function in vegan) and fitted these distances as a response variable in LMMs. All other model details then followed the same as for ‘chemical diversity’ above.

#### Bumblebee Infection

To test how 3-CQA affected *Crithidia* infection, we used a generalised linear mixed model in SAS version 9.4 with *Crithidia* cells per mL^-1^ (log x+1 transformed) as the response, nectar treatment (control, low or high 3-CQA) as a fixed effect, bee size as a covariate, and bee source colony as a random effect. Tukey’s HSD tests were used for *post hoc* pairwise comparisons. Only five bees total (out of 90 bees) died during the course of the experiment, with two deaths in the control treatment, one in the low 3-CQA treatment, and one in the high 3-CQA treatment. These bees were excluded from statistical analysis. Given that so few bees died during the experiment, and that deaths were spread across all treatments, no analysis of mortality was conducted.

## Results

### Chemical Composition

**FIGURE 1:**
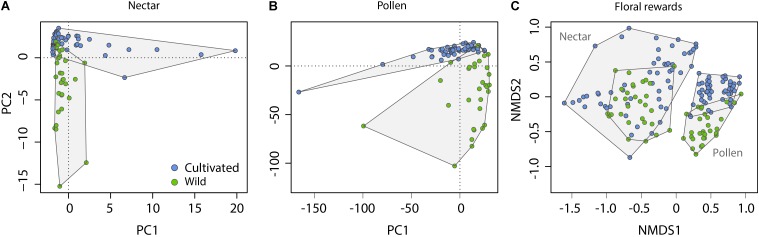
Differentiation of nectar and pollen chemical composition in relation to plant origin (Cultivated vs. Wild). Information on the quantitative concentration of compounds was used to construct PCA ordinations for absolute composition **(A,B)**, whereas relative concentrations of compounds (their percent contribution relative to other compounds in the sample) was used to construct NMDS ordinations for proportional composition **(C)**. Outlying data points in **(A)** are cultivar ‘Spartan’ (four rightmost points) and ‘Northland’; and in **(B)** are cultivar ‘Pioneer.’

Across nectar and pollen samples from wild and cultivated plants, we identified 20 compounds belonging to five chemical classes (12 flavonols, 3 caffeic acid esters, 2 amino acids, 2 flavan-3-ols, and 1 norisoprenoid; **Supplementary Table 2**). Nectar contained 9 compounds, pollen 18, and 7 were shared across the material types. Chemical composition of wild and cultivated plants of *V. corymbosum* was highly distinct for nectar (permutational MANOVA: *F* = 10.5, *p* < 0.001) and pollen (*F* = 15.0, *p* < 0.001; **Figures 1A,B**). Indicator compound analysis revealed six nectar compounds and nine pollen compounds which significantly differentiated these groups (**Table 1**). Of these compounds, a majority (4 out of 6 in nectar; 5 out of 9 in pollen) were more positively associated with wild plants, including the caffeic acid ester 4-*O*-caffeoylshikimic acid (4-CSA). In terms of proportional chemical composition, nectar of wild and cultivated plants was moderately distinct (LMM – NMDS axis 1: *F* = 0.05, *p* = 0.828; NMDS axis 2: *F* = 13.6, *p* = 0.005; **Figure 1C**), whereas large differences existed between groups for pollen (permutational MANOVA: *F* = 30.0, *p* < 0.001; **Figure 1C**).

**Table 1:** ‘Indicator compound analysis’ of nectar and pollen chemical composition in wild and cultivated plants of *Vaccinium corymbosum*.

	Nectar				Pollen			
		
	Wild		Cultivated		Wild		Cultivated	
				
	Value	*p*	Value	*p*	Value	*p*	Value	*p*
**Amino acids**								
Phenylalanine	0.425	0.009	–	–	0.711	0.018	–	–
Tryptophan	–	–	0.421	0.009	–	–	–	–
**Norisoprenoids**								
Roseoside	0.609	0.009	–	–	–	–	–	–
**Phenolic acids**								
4-*O*-caffeoylshikimic acid	0.406	0.009	–	–	–	–	–	–
5-*O*-caffeoylquinic acid	–	–	–	–	–	–	0.378	0.018
**Flavonols**								
Quercitrin	–	–	0.518	0.009	–	–	–	–
Avicularin	0.438	0.009	–	–	–	–	0.399	0.018
Rutin	–	–	–	–	–	–	0.425	0.018
Quercetin-3-*O*-coumaroylhexoside	–	–	–	–	–	–	0.474	0.018
Quercetin-3-*O*-acetylhexoside	–	–	–	–	0.256	0.022	–	–
Kaempferol-3-*O*-rutinoside	–	–	–	–	0.203	0.050	–	–
Quercetin	–	–	–	–	0.538	0.018	–	–
Isorhamnetin-3-*O*-rhamnosylhexoside	–	–	–	–	0.314	0.018	–	–


*Presented are association values (i.e., point-biserial correlation coefficients, bounded between 0 and 1) and their respective *p*-value (derived from permutations, and corrected for multiple testing) for the 13 compounds that had a significantly higher association with one group.*

### Chemical Diversity

**FIGURE 2:**
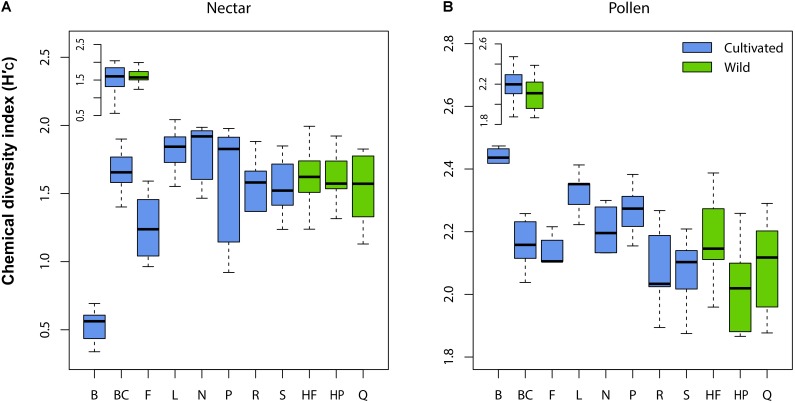
Variation in chemical diversity index (H′_C_) across cultivars and wild populations of *Vaccinium corymbosum* for nectar **(A)** and pollen **(B)**. Significant differences between cultivars were observed for both material types, but not between wild populations. No differences in H′_C_ existed overall between wild and cultivated plants (inset in **A,B**). Abbreviations for cultivars/populations are listed in Methods.

Wild plant nectar consistently lacked the flavonoid glycoside quercitrin, which was abundant in all cultivated plants examined (**Supplementary Table 1**). In contrast, the ester 4-CSA occurred in all wild plants sampled (*n* = 30), but in only 84% of cultivated plants (*n* = 55; **Supplementary Table 2**). As a whole, the chemical diversity index of wild (H′_C_ = 1.59 ± 0.21 SE) and cultivated (H′_C_ = 1.46 ± 0.13) plants did not differ significantly (LMM: *t* = 0.52, df = 9, *p* = 0.618; **Figure 2A** inset). Significant differences in nectar chemical diversity index were, however, seen between individual cultivars (ANOVA: *F*_7,47_ = 11.76, *p* < 0.001; **Figure 2A**), but not between wild populations (*F*_2,27_ = 0.71, *p* = 0.501; **Figure 2A**).

Pollen samples of several wild plants contained four flavonoid glycosides absent in cultivated plants (including glycosides of kaempferol, quercetin, and isorhamnetin). However, because these compounds occurred only sporadically (present in only 7-23% of cases; **Supplementary Table 2**), we found no significant difference in pollen chemical diversity index between wild (H′_C_ = 2.10 ± 0.17 SE) and cultivated (H′_C_ = 2.19 ± 0.22) plants (LMM: *t* = 1.32, df = 9, *p* = 0.220; **Figure 2B** inset). Similar to nectar, pollen chemical diversity index differed significantly across individual cultivars (ANOVA: *F*_7,46_ = 7.94, *p* < 0.001; **Figure 2B**), but not between wild populations (*F*_2,27_ = 2.75, *p* = 0.082).

### Genetic Variation, Correlation, and Heritability

**FIGURE 3:**
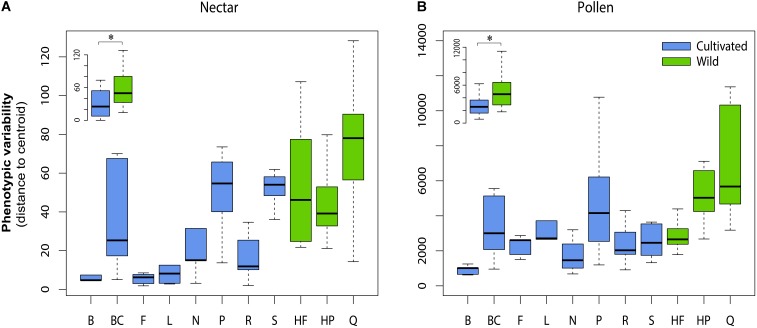
Phenotypic variability in the chemical composition of cultivars and wild populations of *Vaccinium corymbosum* for nectar **(A)** and pollen **(B)**. Phenotypic variability was measured as the Euclidian distance (or dispersion) of plant individuals from their respective cultivar or population multivariate centroid. Significant differences existed overall between wild and cultivated plants for nectar and pollen (inset in **A,B**). Cultivar/population abbreviations are listed in Methods. ^∗^*p* = < 0.05.

For both nectar and pollen, we observed high levels of total genetic variation and broad-sense heritability among cultivars for most chemical traits (**Table 2**). Notable exceptions included total amino acids in pollen, and total phenolic acids and total flavonols in nectar, which exhibited little to no genetic variation. Weak to moderate genetic correlations were observed between nectar traits, in contrast to mostly strong genetic correlation in pollen traits (**Table 2**). Between-material genetic correlations (conducted for the same trait across nectar and pollen) were non-significant for all traits (**Table 2**). While direct comparison of genetic variation between wild and cultivated plants was not possible, differences in the phenotypic variability of chemical composition were significant for both nectar (LMM: *t* = 2.54, *df* = 9, *p* = 0.032; **Figure 3A** inset) and pollen (*t* = 2.59, *df* = 9, *p* = 0.029; **Figure 3B** inset). Cultivated plants exhibited 62.1 and 47.1% less phenotypic variation than wild plants for nectar and pollen, respectively. Thus, more variation could typically be found within single wild populations than in the majority of cultivars examined (**Figures 3A,B**).

**Table 2:** Genetic variation, broad-sense heritability, and genetic correlation of nectar and pollen chemistry across six *Vaccinium corymbosum* cultivars (‘Bonus,’ ‘Friendship,’ ‘Liberty,’ ‘Northland,’ ‘Patriot,’ ‘Reka’ – *n* = 5 plants per cultivar, *n* = 4 for ‘Northland’).

Trait	Material type	Genetic variation (χ^2^)	Heritability (H^2^)	Genetic correlation					
				
				Chemical diversity	Chemical composition	Total Amino acids	Total Phenolic acids	Total Flavonols	Chemical diversity
Chemical diversity	Nectar	19.5^**^	0.70 0.19^**^	-0.04	0.04	0.05	0.03	0.04	0.01
	Pollen	14.6^**^	0.63 0.20^**^						
Chemical composition	Nectar	21.5^**^	0.73 0.18^**^	0.41	0.17	0.76^†^	0.76^†^	0.86^†^	0.61^†^
	Pollen	34.2^**^	0.85 0.14^**^						
Total Amino acids	Nectar	22.6^**^	0.74 0.17^**^	-0.30	-0.45	-0.11	0.77^†^	0.89^†^	0.60^†^
	Pollen	2.4	0.25 0.18^**^						
Total Phenolic acids	Nectar	2.1	0.23 0.17^*^	-0.49	-0.52	0.35	-0.03	0.87^†^	0.73^†^
	Pollen	14.0^**^	0.63 0.20^**^						
Total Flavonols	Nectar	0.0	0.00 0.09	0.07	0.09	0.004	-0.09	0.06	0.68^†^
	Pollen	34.7^**^	0.86 0.13^**^						
Total Flavan-3-ols	Pollen	7.4^*^	0.47 0.21^**^						


*Magnitude of genetic variation is indicated by the chi-squared statistic (*χ*^2^). Within-material genetic correlation is given above and below the diagonal for pollen and nectar, respectively, and between-material correlation on the diagonal. Values for total Flavan-3-ols are presented for pollen only, as this compound class was not identified in nectar. ^†^statistical support inferred from Bayesian 95% credible intervals. ^∗^*p* = < 0.05; ^∗∗^*p* = < 0.001.*

### Bumblebee Infection

**FIGURE 4:**
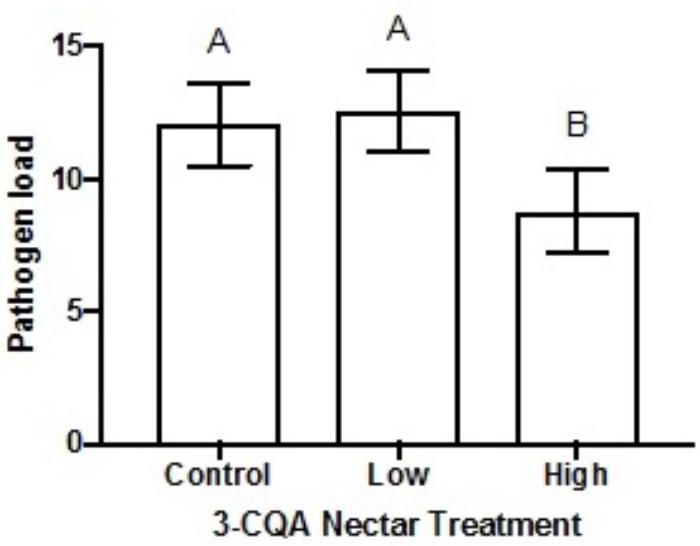
Mean pathogen load of *Crithidia* (log-transformed *Crithidia* per mL + 1) in *Bombus impatiens* workers ( ± SEM) given different dietary concentrations of 3-*O*-caffeoylquinic acid (3-CQA). The control treatment contained 30% sucrose solution. The low treatment corresponded to 2.8 μM ( = 1 ppm) 3-CQA dissolved in 30% sucrose solution, and the high treatment to 56.5 μM ( = 20 ppm) dissolved in 30% sucrose solution. Different upper-case letters above bars indicate statistically significant differences at *p* < 0.05.

Following dietary consumption of 3-CQA by bumble bees parasitised with the gut pathogen *Crithidia*, higher concentrations of 3-CQA significantly reduced infection (*F*_2,79_ = 4.15, *p* = 0.020). Pathogen load was on average reduced by 27% in the high 3-CQA treatment, as compared to a sucrose control or low 3-CQA (**Figure 4**). The covariate of bee size was not related to infection (*F*_1,80_ = 0.03, *p* = 0.856).

## Discussion

Our study provides the first evidence that crop domestication can significantly alter floral reward chemistry, and that such alteration can potentially affect pollinator health via changes in pathogens. While several aspects of nectar and pollen chemistry differed markedly between cultivated and wild plants, this pattern did not hold for all chemical traits, indicating that some traits appeared robust to the domestication process. Of the four hypotheses initially posed, we found at least partial evidence to support each, as discussed in the following sections.

### Domestication Alters Chemical Composition and Diversity of Floral Rewards

As hypothesised, wild and cultivated plants of *V. corymbosum* were differentiated in terms of nectar and pollen chemical composition. However, only pollen had reduced chemical diversity due to domestication; cultivated pollen lacked four flavonoid glycosides which were found in wild pollen. Of the 13 nectar and pollen compounds identified as underlying this differentiation, a majority were positively associated with (or more abundant in) wild plants. However, some examples of the opposite pattern (i.e., greater association with cultivated plants) were also observed for most compound classes (amino acids, phenolic acids, and flavonols), indicating that these differences did not follow a fixed biosynthetic pattern. Flavan-3-ols (catechin and epicatechin, identified only in pollen) did not specifically associate with either group. Of the compounds that differentiated wild or cultivated plants, most did so only for a single material (nectar or pollen). Only two compounds did so for both; phenylalanine showed a consistent pattern across nectar and pollen (positively associated with wild plants for both), whereas the flavonol avicularin showed opposite patterns in nectar and pollen (positive associations with wild plant nectar and cultivated plant pollen).

These results indicate that although domestication effects on the chemical composition and diversity of floral rewards were clear, it is difficult to make *a priori* predictions about which compounds (or compound classes) are affected and in which reward. This difficulty is surprising given the excellent knowledge of the domestication process for highbush blueberry (Hancock and Siefker, 1982; Gough, 1993; Ehlenfeldt, 1994, 2009), in which cultivar provenances, and the characteristics selected for by breeders, are unambiguously known. Selection has typically targeted fruit quality traits such as size, colour, firmness, flavour, and in more recent times, antioxidant content (Kalt et al., 2001). Among various antioxidants, blueberries are a rich source of chlorogenic acid (3-CQA) and anthocyanins, and these compounds correlate with each other in fruit across a wide range of blueberry cultivars (Yousef et al., 2016). Given ongoing selection for rich fruit colour – and by extension 3-CQA – it is reasonable to expect that other caffeic acid esters should increase in floral rewards with domestication. While our results indicated this is the case for 5-CQA in pollen, domestication instead appears to have reduced expression of 4-CSA in nectar. This finding also suggests that fruit chemistry is probably not an accurate proxy for floral reward chemistry. As a whole, given its general unpredictability, we suggest that floral reward chemistry deserves to be monitored in its own right as part of the crop improvement process for blueberry, and possibly entomophilous crops more generally.

### Opportunities and Challenges for Breeding

The majority of nectar and pollen traits examined in cultivars exhibited significant genetic variation and broad-sense heritability, as predicted. The existence of genetic variation in traits is an important prerequisite for selective breeding. In principle, its occurrence offers breeders the potential to fine-tune floral reward chemistry, should such a need be identified. However, genetic correlations (or pleiotropy) may otherwise complicate this process (Luby and Shaw, 2009), and mean that selection cannot be precisely targeted to a trait without causing correlated responses in others, regardless of whether these are desired or not. However, based on two lines of evidence, our findings suggested that different genetic architectures underlie most nectar and pollen chemical traits examined. First, large differences in genetic variation existed for some of the same chemical traits in nectar and pollen; and, second, a majority of within- and between-material genetic correlations were weak or insignificant. Both findings suggest differential genetic regulation of these particular traits, and that pleiotropy does not impose a major constraint. Several pollen chemical traits were exceptions, however, in which large within-material genetic correlations indicate that selection for one trait cannot be achieved independently of others.

Other agronomic metrics of concern to plant breeders include trait and cultivar stability. Here, partitioning phenotypic variance into its total genetic and environmental components allowed us to calculate broad-sense heritability (H^2^). H^2^ also serves as a relative measure of trait stability; low values indicate that most phenotypic variance is the result of environmental plasticity. Somewhat contrary to the prevailing view of nectar traits as particularly plastic (Mitchell, 2004; Parachnowitsch et al., 2018), nectar H^2^ values were mostly high, and generally equivalent to those of pollen. However, total flavonols in nectar were especially unstable (i.e., subject to large environmental variation), as opposed to possessing high stability in pollen. ‘Bonus’ and ‘Friendship’ were the most stable cultivars, based on low variability in their multivariate chemical composition of nectar and pollen. These cultivars hence represent good candidates for cultivation, should stability in floral reward chemistry be an important criterion. Interestingly, the effect of interspecific breeding (to produce hybrid or introgressed cultivars) on stability was not consistent. ‘Friendship’ (a natural wild-collected hybrid between highbush and lowbush blueberry) was one of the most compositionally stable cultivars, whereas ‘BlueCrop’ and ‘Patriot’ (which consist of a 6.4 and 28% genetic contribution from lowbush blueberry, respectively (Lobos and Hancock, 2015) – **Supplementary Table 1**) were the least stable.

Although we could not directly assess the genetic merit of the wild populations sampled, these likely represent good sources of genetically diverse germplasm. The cultivated genepool of *V. corymbosum* has not suffered genetic erosion to the same extent as more distantly domesticated crops, although a narrowing in diversity has nonetheless occurred in recent times (Boches et al., 2006). As discussed, we found little to no genetic variation for a minority of nectar and pollen traits examined, which limits their breeding potential. Given this situation, collection of wild germplasm with the aim to enhance genetic variation in floral reward chemistry could be a desirable and feasible prospect.

### Impacts of Domestication on Pollinator Health

There was evidence to support our hypothesis that domestication effects on antimicrobial compounds in nectar (i.e., 4-CSA in *V. corymbosum*) could impact pollinator health via changes in pathogens. We found concentration-dependent effects of a nectar secondary compound on the gut pathogen *Crithidia*. Low levels of 3-CQA (as a chemically equivalent proxy for 4-CSA at its typical levels in cultivated plants) had no effects on pathogen counts, while high concentrations (within the natural range for 4-CSA in wild plants) significantly reduced *Crithidia* relative to low and control solutions. Our results extend prior research documenting the *in vivo* medicinal value of some secondary metabolites against *Crithidia* (Manson et al., 2010; Richardson et al., 2015). These effects may reflect the effects of these compounds on the bee immune system rather than direct toxicity to parasite cells, given that up to 2500 ppm 3-CQA had no direct effects on *Crithidia* growth in cell culture (Palmer-Young et al., 2016). Moreover, our results are consistent with other studies showing concentration-dependent effects of plant secondary metabolites on insects and their pathogens (Hunter and Schultz, 1993; Cory and Hoover, 2006; Mao et al., 2013; Palmer-Young et al., 2017). Future work should address whether high dietary consumption of 3-QCA or 4-CSA would benefit bee health via reduced pathogen load in natural settings, and whether there are sublethal costs of consuming caffeic acid esters over short and long time-periods. Nonetheless, utilising wild germplasm to breed new cultivars with enhanced levels or profiles of nectar antimicrobials may hold potential.

Beyond effects on pathogens, other facets of pollinator physiology, ecology, and behaviour are also likely influenced by domestication effects on floral reward chemistry. We observed significantly higher associations of the amino acid phenylalanine with wild plant nectar and pollen. The average concentration of this amino acid in wild plant nectar was nearly 4.5 times that of cultivated plants, and nearly 11 times that of cultivated plants for pollen (**Supplementary Table 2**). Phenylalanine is one of several essential amino acids in bee diets (de Groot et al., 1953). However, arguably its most important function is its strong phagostimulatory quality to bees (Inouye and Waller, 1984; Petanidou et al., 2006), believed to enhance nectar taste (Gardener and Gillman, 2002), and influence bee foraging preferences and behaviour at the community scale (Petanidou, 2007). Reduced phenylalanine levels in the nectar and pollen of cultivated *V. corymbosum* suggest large potential ramifications for pollinator attraction and crop pollination success. For instance, several highbush cultivars are relatively unattractive to bees (Filmer and Marucci, 1964; Huber, 2016). The reasons for this are unknown, but result in the need for ‘saturation pollination’ with honey bees (Gough, 1993). A diminished phagostimulatory quality of floral rewards provides one possible explanation for these observations. This hypothesis warrants further investigation.

### Future Research Directions

The potential repercussions of crop domestication for pollinators, mediated through floral reward chemistry, have hitherto remained unexamined for animal-pollinated crops. Our findings on highbush blueberry suggest this topic warrants much wider attention for crops more generally. We therefore suggest future research in four complementary areas.

#### Scale and Trends of Domestication Effects

Establishing the overall scale and specific trends in domestication effects on floral reward chemistry across different types of crop plant would be of great or considerable interest, from both a fundamental and applied perspective. Is alteration of floral reward chemistry a typical outcome of domestication? Does the magnitude of effect depend on the identity of the crop organ(s) artificially selected, as is the case for effects on plant resistance (Whitehead et al., 2017)?

#### Consequences for Crop Pollinators

While the differential attractiveness of certain crop cultivars to pollinators is a well-known phenomenon (Henning et al., 1992; Klatt et al., 2013; Huber, 2016), to what extent could this be related to altered floral reward chemistry, potentially acting in concert with other floral traits? Conversely, could domestication have served to improve floral rewards, such as by reducing toxic or deterrent compound levels [analogous to the response of nectar toxins to plant invasion (Egan et al., 2016; Tiedeken et al., 2016)], and/or boosting levels of attractive or beneficial ones (e.g., pertaining to nutrition, disease resistance, behaviour, phagostimulation)? Are acute impacts of altered floral reward chemistry readily detectable for pollinators, or are negative effects more likely to be chronic, or only apparent in a context-specific manner such as due to disease (de Roode et al., 2013)?

#### Mechanistic Understanding of Change

Elucidation of the physiological and genetic mechanisms through which domestication can alter trait function would permit a deeper understanding of this process in crops. For instance, are some compound classes more likely to be affected than others? Does floral reward chemistry usually show independent genetic regulation, or genetic constraints, which may render it less or more robust to alteration than other plant tissues?

#### Breeding Applications

As has been recognised for other nectar-related traits (Prasifka et al., 2018), tailoring nectar and pollen chemistry for enhanced attraction and functional benefits for pollinators could represent a promising future direction for crop improvement and selective breeding. What are the practical implications for breeding? Can such benefits be unambiguously demonstrated in field settings? What role could wild germplasm play in helping to restore floral rewards which show diminished chemical function, as for other mutualism-related chemical traits (Stenberg et al., 2015)?

#### Conclusion

As a whole, progress on the above areas would offer great insight into crop domestication effects on floral reward chemistry, and the implications and potential breeding solutions for pollinators and crop pollination.

## Data availability

All datasets and R code used in this study will be made publicly available on Figshare.

## Author Contributions

LA, RI, PS, and PE conceived and designed the research. IF and PS performed the chemical analyses. PE analysed the data. PE wrote the manuscript with input and contributions from all authors.

## Conflict of Interest Statement

The authors declare that the research was conducted in the absence of any commercial or financial relationships that could be construed as a potential conflict of interest.
