# Supplementary material for: Crop Domestication Alters Floral Reward Chemistry With Potential Consequences for Pollinator Health
Source: Front Plant Sci. 2018 Sep 26;9:1357. doi: 10.3389/fpls.2018.01357 (PMC6169423; doi:10.3389/fpls.2018.01357)
Supplement: Supplementary file 1 [file Table_1.docx]

**Supplementary Table 1.** Genetic origin of the examined blueberry cultivars. Cultivars represent either pure lines derived from *Vaccinium corymbosum* (VC), or interspecific crosses between *V. corymbosum* and *V. angustifolium* (VA).

| Cultivar | Code | Species origin  (% genetic contribution) |
| --- | --- | --- |
| ‘BlueCrop’ | BC | VC (93.6) x VA (6.4) * |
| ‘Bonus’ | B | VC (100) |
| ‘Friendship’ | F | VC (?) x VA (?) ^†^ |
| ‘Liberty’ | L | VC (100) |
| ‘Northland’ | N | VC (100) |
| ‘Patriot’ | P | VC (72) x VA (28) * |
| ‘Reka’ | R | VC (100) |
| ‘Spartan’ | S | VC (100) |

* data sourced from Lobos and Hancock (2015) and references therein

^†^ “suspected natural hybrid of *V. angustifolium* and *V. corymbosum*” (Gough, 1993),

although in phenotype, plants are thought to more closely resemble *V. corymbosum*

(Stang et al., 1990).

**References:**

Gough, R.E. (1993). *The highbush blueberry and its management.* CRC Press.

Lobos, G.A., and Hancock, J.F. (2015). Breeding blueberries for a changing global environment: a review. *Frontiers in plant science* 6**,** 782.

Stang, E.J., Dana, M.N., Weis, G.G., and McCown, B.H. (1990). 'Friendship' blueberry. *HortScience* 25(12)**,** 1667-1668.
