# Supplementary material for: Crop Domestication Alters Floral Reward Chemistry With Potential Consequences for Pollinator Health
Source: Front Plant Sci. 2018 Sep 26;9:1357. doi: 10.3389/fpls.2018.01357 (PMC6169423; doi:10.3389/fpls.2018.01357)
Supplement: Supplementary file 2 [file Table_2.docx]

**Supplementary Table 2.** Identity, mean concentration, and frequency of occurrence of primary and secondary metabolites determined from the floral rewards of wild and cultivated *Vaccinium corymbosum* plants.

| Compound | Nectar | | | | |  | Pollen | | | | |
| --- | --- | --- | --- | --- | --- | --- | --- | --- | --- | --- | --- |
|  | Wild (n = 30) | |  | Cultivated (n = 55) | |  | Wild (n = 30) | |  | Cultivated (n = 54) | |
|  | Mean µM  (± SE) | % plants present |  | Mean µM  (± SE) | % plants present |  | Mean µM  (±SE) | % plants present |  | Mean µM  (± SE) | % plants present |
| **Amino acids** |  |  |  |  |  |  |  |  |  |  |  |
| Phenylalanine | 28.8 (± 5.0) | 100 |  | 6.6 (± 1.1) | 100 |  | 3588 (± 650) | 97 |  | 327 (± 37) | 100 |
| Tryptophan | 2.6 ± (0.6) | 100 |  | 19.0 (± 3.6) | 98 |  | 447 (± 90) | 100 |  | 686 (± 53) | 100 |
| **Norisoprenoids** |  |  |  |  |  |  |  |  |  |  |  |
| Roseoside | 70.5 (± 9.7) | 97 |  | 15.9 (± 1.6) | 98 |  |  |  |  |  |  |
| **Phenolic acids** |  |  |  |  |  |  |  |  |  |  |  |
| 4-*O*-caffeoylquinic.acid | 13.6 (± 3.7) | 77 |  | 6.8 (± 1.7) | 84 |  | 5.4 (± 19.0) | 100 |  | 88.9 (± 8.97) | 100 |
| 5-*O*-caffeoylquinic.acid | 12.2 (± 2.1) | 80 |  | 30.2 (± 9.7) | 91 |  | 362 (± 100) | 100 |  | 879 (± 109) | 100 |
| 4-*O*-caffeoylshikimic.acid | 20.6 (± 5.1) | 100 |  | 4.9 (± 1.1) | 84 |  |  |  |  |  |  |
| **Flavonols** |  |  |  |  |  |  |  |  |  |  |  |
| Hyperoside | 20.7 (± 3.4) | 97 |  | 38.4 (± 10.9) | 98 |  | 3334 (± 534) | 100 |  | 3736 (± 444) | 100 |
| Quercitrin | - | 0 |  | 7.0 (± 1.12) | 100 |  | 870 (± 194) | 80 |  | 998 (± 185) | 100 |
| Avicularin | 30.4 (± 5.0) | 100 |  | 12.7 (± 2.5) | 98 |  | 1426 (± 340) | 40 |  | 1675 (± 284) | 100 |
| Rutin |  |  |  |  |  |  | 988 (± 254) | 63 |  | 1342 (± 166) | 96 |
| Quercetin-3-*O*-coumaroylhexoside |  |  |  |  |  |  | 412 (± 58) | 100 |  | 1463 (± 225) | 100 |
| Quercetin-3-*O*-acetylhexoside |  |  |  |  |  |  | 383 (± 113) | 17 |  | - | 0 |
| Kaempferol-3-*O*-rutinoside |  |  |  |  |  |  | 366 (± 174) | 13 |  | - | 0 |
| Quercetin |  |  |  |  |  |  | 769 (± 87) | 100 |  | 488 (± 43) | 100 |
| Isorhamnetin-3-*O*-rhamnosylhexoside |  |  |  |  |  |  | 937 (± 381) | 23 |  | - | 0 |
| Quercetin-3-O-pentoside |  |  |  |  |  |  | 1237 (± 231) | 100 |  | 1971 (± 200) | 100 |
| Q.3.O..rutinoside..O.met |  |  |  |  |  |  | 452 (± 335) | 10 |  | - | 0 |
| kaempferol.O..coumaroyl |  |  |  |  |  |  | 916 (± 155) | 100 |  | 855 (± 119) | 100 |
| **Flavan-3-ols** |  |  |  |  |  |  |  |  |  |  |  |
| Catechin |  |  |  |  |  |  | 1663 (± 267) | 100 |  | 3440 (± 606) | 100 |
| Epicatechin |  |  |  |  |  |  | 1286 (± 526) | 100 |  | 874 (± 92) | 100 |
